# Combining Sandblasting, Alkaline Etching, and Collagen Immobilization to Promote Cell Growth on Biomedical Titanium Implants

**DOI:** 10.3390/polym13152550

**Published:** 2021-07-31

**Authors:** Chia-Fei Liu, Kai-Chun Chang, Ying-Sui Sun, Diem Thuy Nguyen, Her-Hsiung Huang

**Affiliations:** 1 Department of Dentistry, National Yang Ming Chiao Tung University, Taipei 112, Taiwan; chiafeiliu@nycu.edu.tw (C.-F.L.); rosenguyen7196@nycu.edu.tw (D.T.N.); 2 Institute of Oral Biology, National Yang Ming Chiao Tung University, Taipei 112, Taiwan; karineous09@gmail.com; 3 School of Dental Technology, Taipei Medical University, Taipei 110, Taiwan; yingsuisun@tmu.edu.tw; 4 Department of Bioinformatics and Medical Engineering, Asia University, Taichung 413, Taiwan; 5 Department of Medical Research, China Medical University Hospital, China Medical University, Taichung 404, Taiwan; 6 Department of Stomatology, Taipei Veterans General Hospital, Taipei 112, Taiwan; 7 Department of Education and Research, Taipei City Hospital, Taipei 103, Taiwan

**Keywords:** cell growth, titanium implant, surface modification, sandblasting, alkaline etching, type I collagen immobilization

## Abstract

Our objective in this study was to promote the growth of bone cells on biomedical titanium (Ti) implant surfaces via surface modification involving sandblasting, alkaline etching, and type I collagen immobilization using the natural cross-linker genipin. The resulting surface was characterized in terms topography, roughness, wettability, and functional groups, respectively using field emission scanning electron microscopy, 3D profilometry, and attenuated total reflection-Fourier transform infrared spectroscopy. We then evaluated the adhesion, proliferation, initial differentiation, and mineralization of human bone marrow mesenchymal stem cells (hMSCs). Results show that sandblasting treatment greatly enhanced surface roughness to promote cell adhesion and proliferation and that the immobilization of type I collagen using genipin enhanced initial cell differentiation as well as mineralization in the extracellular matrix of hMSCs. Interestingly, the nano/submicro-scale pore network and/or hydrophilic features on sandblasted rough Ti surfaces were insufficient to promote cell growth. However, the combination of all proposed surface treatments produced ideal surface characteristics suited to Ti implant applications.

## 1. Introduction

The use of biomaterials in implants is a promising development in tissue rehabilitation. Titanium (Ti) is particularly useful in implant applications, thanks to its good corrosion resistance, mechanical properties, and biocompatibility [1,2,3]. However, the bioinert surface nature of Ti and its alloys has been implicated in primary implant failure, due to insufficient osseointegration within the first few months [2,4,5]. This has prompted widespread research in bioactive surface modification techniques aimed at enhancing the bioaffinity of Ti [4,6].

Researchers have developed a range of biomimetic coatings, such as hydroxyapatite and bioglass [2,7], as well as methods to promote the formation of bioactive phases via alkaline heat treatment, H_2_O_2_ treatment, and direct oxidation in air [2]. Another approach involves the sandblasting of Ti surfaces using alumina (Al_2_O_3_) particles to facilitate osseointegration by increasing surface roughness [8,9,10,11]: this approach has also been shown to improve the interfacial shear strength between Ti and the surrounding bone. Furthermore, sandblasting has been implemented in conjunction with other techniques, such as acid etching, to further enhance the benefits of surface modification [10,12]. Research studies have generated three-dimensional micro- and submicro-scale pore structures by combining these methods with anodization [13] or micro-arc oxidation [14]. Note, however, that many of these schemes fail to induce cell differentiation into osteoblasts, due largely to a lack of exogenous soluble factors [15]. Bone morphogenetic protein-2 (BMP-2) has been used as a surface coating layer to overcome this issue [12,16]; however, BMP-2 is costly and its application is hampered by the short half-life in vivo [17].

Type I collagen has been proposed as an alternative to BMP-2, due to its superior biocompatibility, low immunogenicity, and widespread use as a tissue replacement material in medical applications [18]. Collagen accounts for roughly 95% of the organic matrix in bone tissue and plays important roles in a variety of osteoblast activities [19], such as tissue repair through the activation of platelets via the integrin α2β1-binding domain [20]. These molecules also function as mediators of osteoblast cell responses [21], including in proliferation and migration during the healing process [22,23,24] as well as in secretion of the extracellular matrix (ECM) and osteoblast differentiation [21].

A variety of methods have been devised to promote the crosslinking of collagen to reduce the rate of enzymatic degradation in vitro [25]. Unfortunately, physical scaffolds [25,26] lack mechanical integrity, and many chemical crosslinkers [27] are potentially cytotoxic [25,28]. This has prompted widespread interest in genipin, a natural crosslinking agent obtained from the fruit of Genipa Americana [25,26,28] with toxicity levels 10^4^ times lower than that of glutaraldehyde [26,28]. The effects of genipin on collagen adhesion have also been shown to last for more than a few days [29].

In the current study, we created a hydrophilic surface with a nano/submicro-scale pore network (via alkaline etching) for use in conjunction with type I collagen immobilization to promote cell growth on sandblasted rough Ti surfaces. The resulting Ti surfaces were then characterized in terms of morphology, roughness, wettability, and chemistry. The proposed surface modification scheme proved highly effective in promoting the adhesion, proliferation, initial differentiation, and mineralization of human bone marrow mesenchymal stem cells (hMSCs).

## 2. Materials and Methods

### 2.1. Sample Preparation

All Ti specimens were derived from grade IV Ti discs (Ultimate Materials Technology Co., Ltd., Hsinchu, Taiwan) with a diameter of 16 mm and a thickness of 1 mm after grinding using #1200 silicon carbide sandpapers. The Ti specimens were first roughened by vertical sandblasting treatment (pressure 4 bar) using Al_2_O_3_ particles (size 120 μm) with a distance 10 cm for 10 s with the aim of promoting mechanical interlocking between the Ti implants and surrounding bone. Specimens that underwent sandblasting alone were designated SBTi. Some of the SBTi specimens were then submerged in NaOH solution (5 M) for 24 h to generate surface hydroxyl (OH) groups (designated SBTiOH). Some of the SBTiOH specimens underwent incubation in phosphate buffered saline (PBS) containing 0.01% genipin (>98% C_11_H_14_O_5_, Challenge Bioproducts Co., Touliu, Taiwan) at 37 °C for 24 h (designated SBTiG). Note that this process was meant to promote chemical bonding between negatively charged hydroxyl groups on SBTiOH surfaces and aldehyde (CO) groups on the genipin structure. Some of the SBTiG specimens were immersed in PBS containing 0.1% type I collagen from calf skin (C9791, Sigma-Aldrich, Saint Louis, MO, USA) at 4 °C for 24 h (designated SBTiGC). Note that in this process, N-terminals of collagen reacted with CO-bonded surfaces of SBTiG to generate amide bonds. All the abovementioned surface treatments were carried out in a laboratory with controlled temperature (~25 °C) and humidity (~70%).

### 2.2. Surface Characteristics

The surface morphology of the test samples was examined using a field emission-scanning electron microscope (FE-SEM; JSM7600, JEOL Ltd., Tokyo, Japan). Surface roughness values (arithmetic average roughness (Sa) and root-mean-square height (Sq)) were obtained using a 3D profilometer (Profilm 3D, KLA-Filmetrics, San Diego, USA) with a scanning area of 230 µm × 150 µm and Gaussian filter size (cutoff) of 25 μm. The sessile solid drop method was used to evaluate the wettability (or hydrophilicity) and surface energy. Deionized water and diiodomethane were used as representative polar and non-polar liquids. A contact angle goniometer (100SB, Sindatek, New Taipei City, Taiwan) was used to capture the side view of the liquid droplet on the sample surface, and then the contact angle was measured. The corresponding software Magic Droplet was used to calculate the surface energy using the Owens–Wendt method [30]. The abovementioned measurements were averaged from measurements performed in triplicate. The sample size for each measurement was 5. Functional groups on the surface of test samples were also analyzed using attenuated total reflection-Fourier transform infrared spectroscopy (ATR-FTIR; FT-IR Spectrometer Frontier, PerkinElmer Inc., Waltham, MA, USA).

### 2.3. Cell Adhesion

Cell adhesion tests were performed using hMSCs transduced via retroviral delivery using the gene for green fluorescent protein (GFP). The GFP-labeled hMSCs were seeded on test samples (10^5^ cell/disc) in a Gibco Dulbecco’s Modified Eagle’s Medium (DMEM; D6421, Merck KGaA, Darmstadt, Germany) including 5% fetal bovine serum and 10% horse serum (all from Sigma-Aldrich). Following incubation for 24 h, cells adhesion to test samples was imaged in situ using a fluorescence microscope. In the parallel testing, the adherent cells on test samples were fixed, dehydrated, and then completely dried using a critical point dryer. Afterwards, the samples were coated with a platinum thin film for the observation of cell adhesion morphology via FE-SEM.

### 2.4. Cell Proliferation

hMSCs proliferation was examined using 3-(4,5-dimethylthiazol-2-yl)-2,5-diphenyltetrazolium bromide (MTT) (Sigma-Aldrich) assay. Briefly, cells were seeded on test samples (10^4^ cell/disc) and cultured for one week before being incubated in medium containing MTT under 5% CO_2_ at 37 °C for 4 h. Isopropanol was used to dissolve purple formazan, and observations were performed at days 1, 4, and 7 using a microplate photometer to measure absorbance at a wavelength of 570 nm. Higher optical density (OD) values indicate superior cell viability.

### 2.5. Cell Differentiation and Mineralization

The protein expression level of early stage marker osteopontin (OPN) on cell differentiation on test samples was analyzed with human OPN DuoSet enzyme-linked immunosorbent assay (ELISA) kit (DY 1433, R&D systems, Minneapolis, MN, USA) at days 7 and 14. The experimental details were carried out in accordance with the manufacturer’s instructions. Furthermore, cell mineralization on test samples was qualitatively and quantitatively analyzed by the formation of calcium compounds using Alizarin red S (Sigma-Aldrich) staining. hMSCs were cultured at a density of 10^4^ cell/disc in normal culture medium for one day prior to immersion in an osteogenic medium containing DMEM supplemented with 50 μg/mL ascorbic acid, 10 mM β-glycerophosphate, and 10^−8^ M dexamethasone (all from Sigma-Aldrich). The osteogenic medium was changed every 48 h. After osteogenic incubation for 7 or 14 d, cells were sequentially fixed and stained using Alizarin red S (2%) at room temperature for 20 min. Mineralization of the ECM resulted in large deposits of calcium and phosphorus ions. The stained cells were immersed in cetylpyridinium chloride and sodium phosphate at room temperature under shaking for 1 h. OD values were recorded using a microplate photometer at a wavelength of 540 nm, where higher OD values indicate more extensive cell mineralization. Note that the OD values of the blank groups (the same surface treatment without cell culture) were below 0.01 (nearly negligible) and were subtracted from the measured OD values of the corresponding test groups.

### 2.6. Statistical Analysis

Experimental data are presented as mean ± standard deviation (SD). All measurements were performed in triplicate. The sample size of each test group at a specific time point per measurement was 5. One-way analysis of variance (ANOVA) was used to analyze the effect of surface treatment on surface roughness and cell responses with significance at α = 0.05. Tukey’s test was used for pairwise comparisons.

## 3. Results

### 3.1. Surface Characteristics

**Figure 1:**
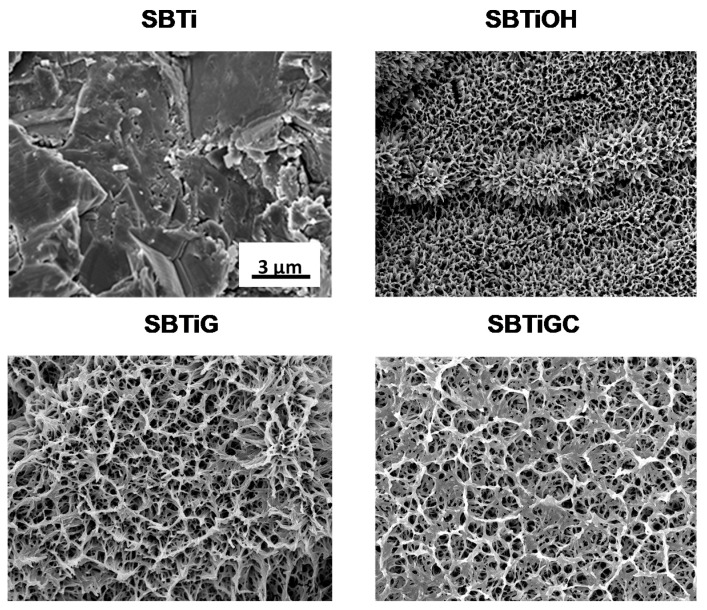
Surface FE-SEM micrographs of test samples.

Figure 1 presents the surface FE-SEM micrographs of SBTi, SBTiOH, SBTiG, and SBTiGC specimens. SBTi specimens presented a rough surface with a topography characterized by undulations. SBTiOH specimens presented a rough surface with a network structure, comprising pores at the nano/submicrometer scale (approximately 100~200 nm). SBTiG and SBTiGC specimens remained a rough topography with nano/submicro-scale pore network, while showing a visible genipin mesh and type I collagen sheet, respectively.

**Figure 2:**
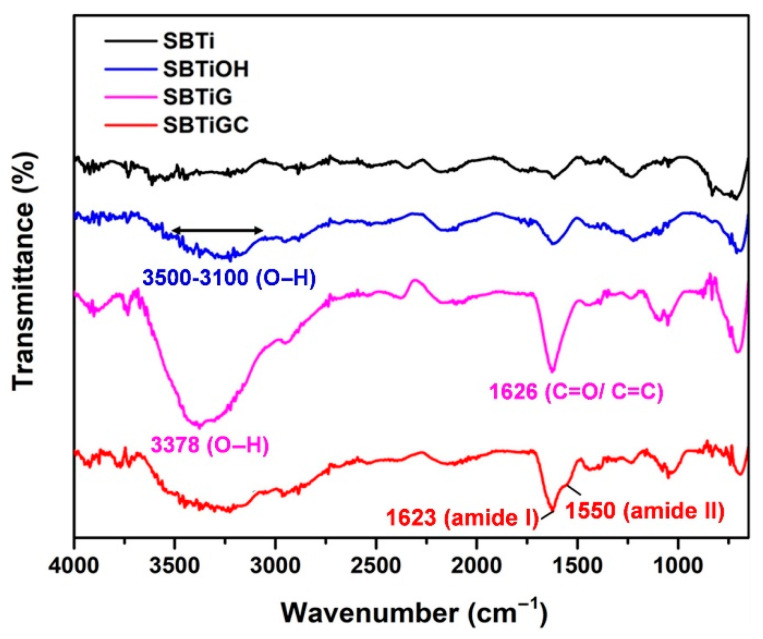
Surface ATR-FTIR spectra of test samples.

The functional groups on Ti specimens with and without surface treatment were analyzed using ATR-FTIR (Figure 2). On SBTiOH specimens, absorption peaks indicative of hydroxyl (O–H) groups were observed at 3100–3500 cm^−1^. The intensity of the absorption peak related to O–H stretching vibration (3378 cm^−1^) on SBTiG was higher than on SBTiOH. The absorption peak at 1626 cm^−1^ on SBTiG can be attributed to the stretching vibration of either the C=O or C=C groups in genipin. We observed the typical amide bands of type I collagen in the spectra of SBTiGC: C=O stretching vibration absorption peak (1623 cm^−1)^ and N–H bending vibration peak (1550 cm^−1^).

As shown in Table 1, surface treatment had significant effect on Sa values (*p* < 0.05), and the corresponding Tukey’s test showed that two different groups, SBTi and (SBTiOH, SBTiG, SBTiGC), were observed. However, surface treatment did not have significant effect on Sq values (*p* > 0.05). Overall, SBTi presented micrometer-scale roughness values slightly exceeding the other specimens (in terms of mean value of Sa or Sq). The overall roughness ranges were 0.87–1.10 μm (Sa) and 1.13–1.27 μm (Sq).

**Figure 3:**
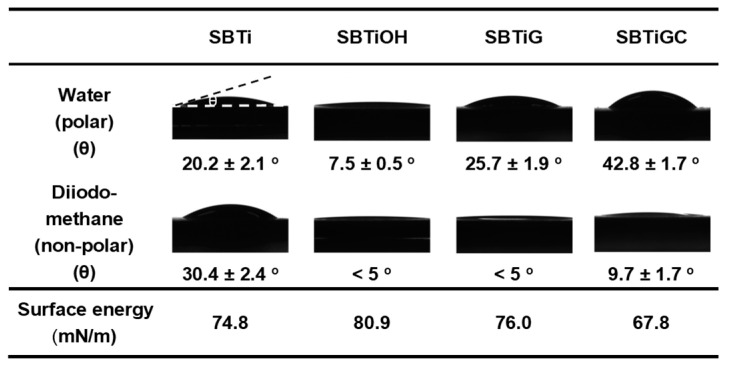
Wettability of test samples, in term of contact angle and surface energy.

Figure 3 presents surface wettability analysis results obtained using contact angle measurements in polar (deionized water) and non-polar (diiodomethane) solvents. The surface contact angle of the SBTi surfaces was 20° in water and 30° in diiodomethane. The surface contact angles of the SBTiOH were less than 8° thanks to the introduction of surface hydroxyl groups. The surface contact angles of SBTiG and SBTiGC in non-polar solvent were below 10°; however, the contact angle (roughly 26°) of SBTiG in polar solvent was close to that in specimen treated using sandblasting alone (SBTi). The water contact angle of SBTiGC was 43°. According to the American Society for Testing and Materials (ASTM) D7334-08 specifications, a water contact angle of less than 45° is indicative of a hydrophilic surface. A higher surface energy is also indicative of better wettability. All test samples in the current study were hydrophilic, as indicated by low water contact angle and high surface energy (68–81 mN/m). Overall, alkaline etching produced a superhydrophilic surface; whereas coating genipin and type I collagen still resulted in a hydrophilic surface.

In order to evaluate the depth of genipin and collagen on test samples, X-ray photoelectron spectroscopy (XPS; Sigma Probe, Thermo VG Scientific, East Grinstead, UK) was used to analyze the C1s and N1s spectra as a function of depth on SBTiG and SBTiGC surfaces, respectively (data not shown). The corresponding depth was estimated as the spectrum intensity became less obvious (or flatter). Accordingly, the estimated depth of genipin (in terms of C1s spectrum) and collagen (in terms of N1s spectrum) on SBTiG and SBTiGC, respectively, were approximately 100 nm and 65 nm.

### 3.2. Cell Adhesion

**Figure 4:**
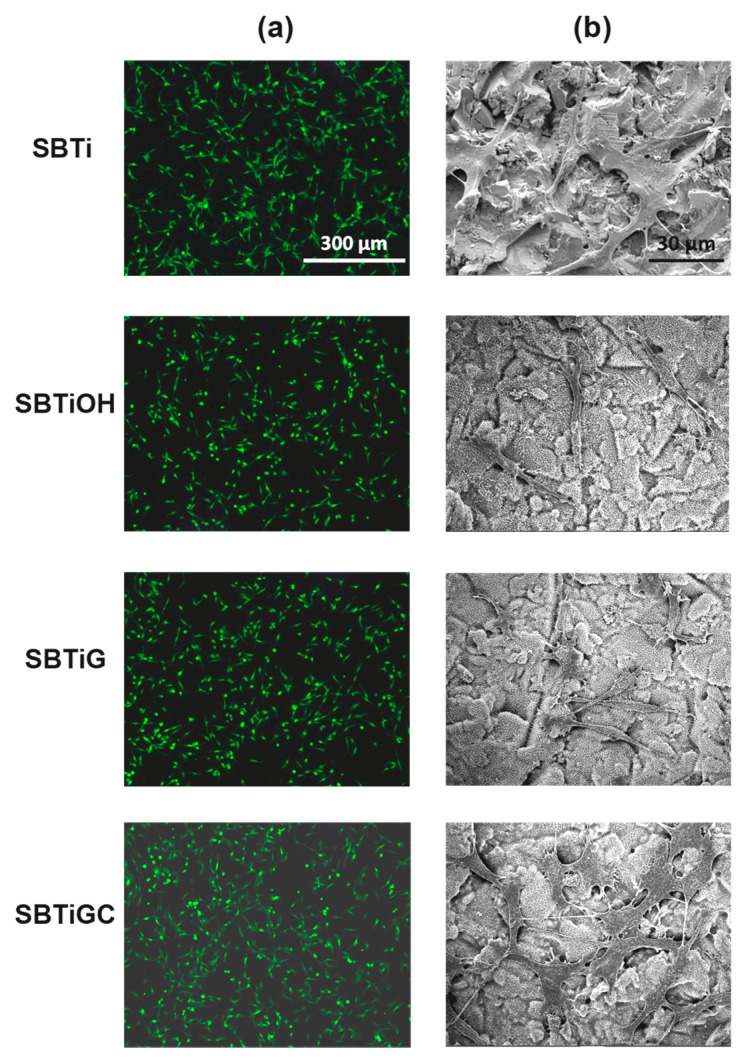
(**a**) Fluorescence microscopy images and (**b**) FE-SEM micrographs indicating cell adhesion of GFP-labeled hMSCs on test samples following an incubation period of 24 h.

We assessed the adhesion performance of GFP-labeled hMSCs incubated on test samples for 24 h. The in situ cell distribution was observed using an upright fluorescence microscope, and adhesion morphology was imaged via FE-SEM (Figure 4). The fluorescence images revealed no significant differences among test samples in terms of cell distribution (Figure 4a). The FE-SEM micrographs revealed that the cell attachment on SBTi and SBTiGC was superior to that on SBTiOH and SBTiG (Figure 4b). Note that cell–cell interactions were more pronounced in the SBTiGC specimen.

### 3.3. Cell Proliferation

**Figure 5:**
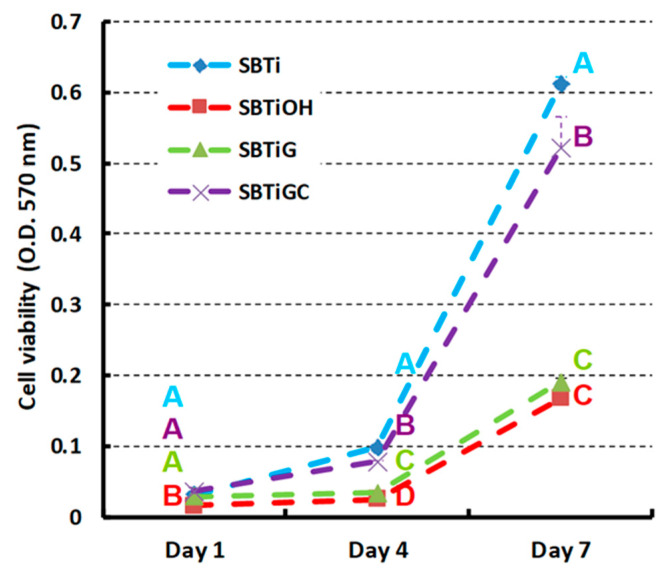
Proliferation of hMSCs on test samples following cell incubation for 7 d (groups with different letters are significantly different (*p* < 0.05)).

Figure 5 presents the cell proliferation on test samples during incubation of 7 d. Surface treatment had a significant effect on cell proliferation at days 1 (*p* < 0.01), 4 (*p* < 0.001), and 7 (*p* < 0.001). The outstanding hMSCs proliferation was achieved by sandblasting (SBTi) and immobilizing type I collagen (SBTiGC), particularly between days 4 and 7; SBTiGC showed slightly lower cell proliferation than SBTi. However, the SBTiOH and SBTiG specimens presented far more modest cell proliferation.

### 3.4. Cell Differentiation and Mineralization

**Figure 6:**
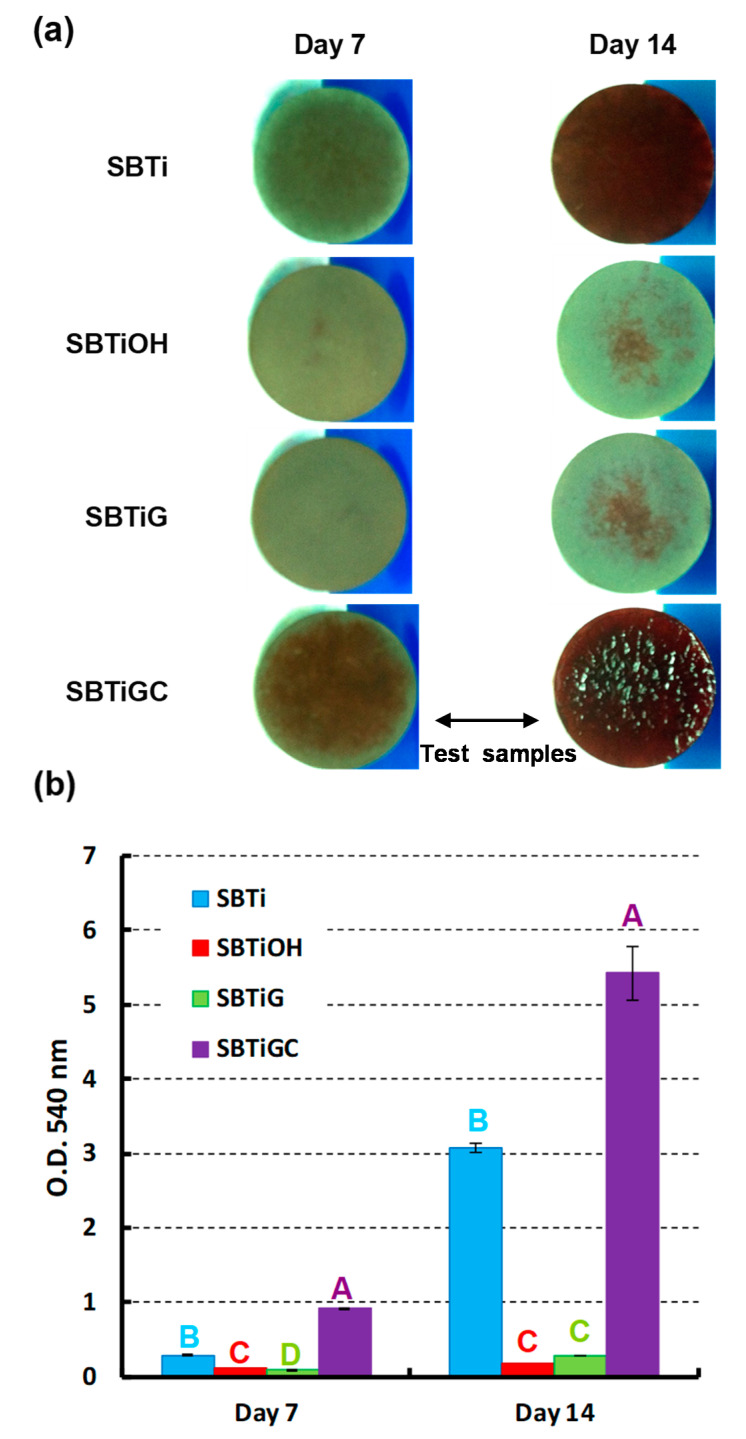
(**a**) Qualitative and (**b**) quantitative data pertaining to mineralization of hMSCs based on Alizarin red S staining analysis following incubation for 7 or 14 d (groups with different letters are Scheme 0.

Table 2 presents ELISA analysis results, indicating the protein expression level of OPN of test samples after cell incubation of 7 and 14 d. Surface treatment had significant effect on OPN expression level at days 7 and 14 (*p* < 0.001). The OPN expression level was significantly higher on SBTi and SBTiGC specimens, particularly SBTiGC at day 14, than on SBTiOH and SBTiG specimens. Figure 6 presents Alizarin red S staining results used to (a) qualify and (b) quantify the mineralization of ECM in hMSCs after incubation of 7 and 14 d. Surface treatment had significant effect on mineralization in hMSCs at days 7 and 14 (*p* < 0.001). After 7- or 14-day incubation in an osteogenic culture, the specimen coated with collagen (SBTiGC) qualitatively and quantitatively outperformed all other specimens (SBTi, SBTiOH, and SBTiG) in terms of cell mineralization. Cells seeded on SBTiOH and SBTiG specimens presented a significantly weak mineralization, which did not vary noticeably throughout the experiment. These results are similar to those for cell adhesion and cell proliferation (Figure 4 and Figure 5).

## 4. Discussion

As shown in Figure 1**,** SBTi specimens presented rough surface undulations, whereas SBTiOH, SBTiG, and SBTiGC specimens presented a nano/submicro-scale network with pores of 100~200 nm on rough surfaces. Previous studies have reported that some nanostructures can mimic the structure of the ECM, thereby enabling the surface to interact with receptors on the cell membrane to affect cell responses [31]. This type of network has also been shown to enhance hydrophilicity and cell responses and to improve biocompatibility [31,32]. In the current study, alkaline etching treatment led to the formation of a nano/submicro-scale pore network on SBTiOH specimens. Coating with genipin (SBTiG) and type I collagen (SBTiGC) did not seriously alter this network feature.

ATR-FTIR analysis results (Figure 2) confirmed the success of Ti surface modification. The broad absorption peaks at 3100–3500 cm^−1^ indicate the presence of hydroxyl groups after alkaline etching treatment [33]. The super hydrophilicity of the TiGOH specimen was primarily based on the presence of surface hydroxyl groups [34]. Note, however, that there was a significant increase in O–H signals after coating the surface with genipin (SBTiG), indicating that alkaline etching in conjunction with genipin immobilization led to the formation of even more O–H groups. Genipin immobilization on SBTiG specimens can be attributed to two functional groups: the carboxymethyl (C=O) group [35] and C=C vibration of the olefin ring [36], as indicated by an absorption peak at 1626 cm^−1^. However, overlapping of the two functional groups made it impossible to detect them separately. After genipin immobilization on SBTiOH surface (SBTiG), although the O–H and C=O/C=C groups increased, the C=O/C=C groups were predominant on the genipin structure instead of the hydrophilic O–H group. Hence, despite the increased O–H group on SBTiG surface (vs. SBTiOH surface), the hydrophilicity of SBTiG was still less than that of SBTiOH due to the covering of C=O/C=C groups on SBTiG surface.

The two main vibration bands of protein in the infrared spectrum are the amide I and amide II bands [33]. Amide I absorption (1600–1700 cm^−1^) arises predominantly from protein amide C=O stretching vibrations, whereas amide II absorption (1500–1550 cm^−1^) arises predominantly from N–H bending [37]. Figure 2 confirmed that both of amide I and amide II were detected on the surface of SBTiGC. After surface treatments on a Ti surface, the roughness and wettability of the surface may change accordingly, leading to effects on cell growth, as will be discussed later.

The results in Table 1 reveal that none of the surface treatments significantly altered surface roughness at the micrometer scale (Sa 0.87–1.10 μm; Sq 1.13–1.27 μm). Rather, they resulted in the formation of a network structure comprising pores of various sizes ranging from the nanoscale to the submicron scale (Figure 1). We expected that these surface treatments would enhance hydrophilicity and thereby promote early cell adhesion [38]. In a previous study [39], an electrochemical anodization treatment was used to create a superhydrophilic surface porosity on sandblasted Ti dental implants for promoting the bone cell growth in vitro and in vivo, with surface roughness values (Sa~1.0 μm; Sq~1.2 μm) similar to those in the current study. The surface Sa value of commercial sand-blasted and acid-etched (SLA) Ti implants is ca. 1.18 μm [40]. In this study, the Sa value of the sand-blasted Ti surface (SBTi) was ca. 1.10 μm, which was close to that of the commercial SLA Ti implants. As for the surface-modified Ti specimens (SBTiOH, SBTiG, and SBTiGC), the Sa values were ca. 0.87–0.93 μm, which were slightly lower than those of the SBTi specimen and commercial SLA Ti implants. We believe that this was due to the slight smoothing effect caused by alkaline etching and/or biomolecule coverage on the SBTi surface.

As shown in Figure 3, the surface treatments proposed in the current study approximately enhanced the surface wettability (or hydrophilicity), except that the water contact angle of SBTiGC was higher than that of other groups even though SBTiGC still remained a hydrophilic surface. The commercial SLA Ti surfaces are usually hydrophobic and have water contact angle 70–120° [40,41]. In this study, the hydrophilicity of the sand-blasted Ti surface (SBTi) was believed to be related to the parameters used for sandblasting process and the laboratory environment. It appears that an increase in surface area made possible by sandblasting was slightly nullified by surface immobilization of type I collagen, which partially covered the surface pores with a corresponding effect on surface wettability. Studies have demonstrated that hydrophilic surfaces can promote cell differentiation and early osseointegration in animal testing [42]. Conversely, one study has reported that surface hydrophilicity has no effect on the adhesion of osteoblasts [43]. It appears that surface wettability alone is insufficient to explain the biocompatibility of materials. The synergetic effects of various surface characteristics on cell growth need to be investigated in the future.

The initial adhesion of cells to biomaterials is crucial to subsequent cell–material interactions and corresponding cell responses, including proliferation and differentiation [44,45]. Figure 4 illustrates the notable spreading shapes of cells adhered to SBTi and SBTiGC specimens. Note that cells adhering to SBTiOH and SBTiG specimens presented the same slender morphology, although both exhibited a hydrophilic surface (with water contact angle of 8–26° and diiodomethane contact angle of less than 5°), which implied that the specimens were ill-suited to hMSCs attachment. By contrast, the hydrophilic surface of SBTi and SBTiGC (with water contact angle of 20~43° and diiodomethane contact angle of 10–30°), was well-suited to cell adhesion. Previous studies have reported that superhydrophilic Ti surfaces enhance cell attachment and/or proliferation [31,46,47]. On the contrary, one recent study reported that superhydrophilic surfaces are not conducive to cell adhesion or growth [48]. These authors stated that the hydrophilicity of the material surface influences the characteristics of the proteins adsorbed from the culture medium, which can have a profound effect on cell attachment. Sequentially, superhydrophilic surfaces inhibit the binding of cell adhesion mediators, thereby hindering cell adhesion [48]. Our results found that SBTiOH and SBTiG specimens had worse cell adhesion morphology than the SBTIGC specimen, although SBTiOH and SBTiG surfaces had better wettability and similar physical properties (morphology and roughness) to the SBTiGC surface. Obviously, only wettability (or hydrophilicity) is insufficient to explain cell adhesion behavior. The synergetic effect of various surface characteristics (morphology, roughness, wettability, and mechanical properties, etc.) on cell adhesion needs to be considered in the future. On the other hand, previous studies have widely reported that nano-scale porosity on Ti and/or Ti alloy surfaces promotes cell adhesion [31,47]. However, the nano/submicro-scale pore networks on SBTiOH and SBTiG surfaces did not positively affect the cell adhesion in this study. The most significant cell–cell interaction was found on the collagen-immobilized SBTiGC surface containing a nano/submicro-scale pore network with acceptable hydrophilicity(water contact angle of 43°). It has been reported that the presence of type I collagen enhances the spreading and filopodia extension of cells on Ti alloy [33,49]. Note that further experiments will be required to determine the underlying mechanism of surface biomolecule immobilization on cell adhesion.

Cell proliferation is a crucial stage in the bone repair process. As shown in Figure 5, cell proliferation was significantly higher on SBTi and SBTiGC specimens than on SBTiOH and SBTiG. This result is consistent with our cell adhesion results (Figure 4). For comparison, the cell proliferation, in terms of OD value, of the untreated Ti surface (ground with #1200 silicon carbide sandpapers) was also performed and listed here (data not shown in Figure 5): 0.029 ± 0.001 at day 1; 0.161 ± 0.015 at day 4; 0.420 ± 0.018 at day 7. Although the untreated Ti specimen showed similar or slightly higher OD values to the SBTi and SBTiGC specimens during the 4-day cell incubation, its OD value became much lower than that of SBTi and SBTiGC at day 7.

Taken together, biomolecule immobilization played a key role in promoting these stages of the cell response. Previous studies have reported that nano/submicro-scale porosity has a positive effect on the cell proliferation [31,50]; however, a similar nano/submicro-scale pore network in the current study (generated via alkaline etching) did not necessarily affect cell adhesion and/or proliferation, as demonstrated by the fact that all the surface-treated specimens (SBTiOH, SBTiG, and SBTiGC) possessed the similar surface characteristics (i.e., pore network feature and roughness). Additionally, the difference in wettability (or hydrophilicity), mainly affected by the surface functional groups, also could not be directly related to the cell response.

As shown in Table 2 and Figure 6**,** SBTiOH and SBTiG specimens did not present substantial OPN expression and ECM mineralization; however, the SBTiGC specimen (with type I collagen) presented excellent initial cell differentiation in terms of OPN expression level and cell mineralization ability. The immobilization of type I collagen on Ti surfaces has attracted considerable attention for its effect on osteoblast survival, cell proliferation, cell differentiation, and matrix mineralization [12,51]. Type I collagen plays a particularly important role in bone mineralization [52]. Overall, cell adhesion and proliferation were very similar in SBTiGC and SBTi specimens; however, mineralization was more pronounced in SBTiGC specimens at 7 and 14 days. Although SBTiGC had a slightly lower cell proliferation than SBTi at day 7 (Figure 5), significantly higher expressions of early stage marker OPN on cell differentiation as well as on ECM mineralization were obtained for the SBTiGC specimen (vs. SBTi). This implies that the combined surface treatments for SBTiGC slightly decreased the cell proliferation (vs. SBTi) and then rapidly accelerated the cell growth to the next stage, i.e., differentiation and mineralization. Similar results, showing the decreasing cell proliferation but increasing cell differentiation and/or mineralization, have been reported elsewhere [39,53]. Our results indicate that even hydrophilic surfaces with a nano/submicro-scale pore network structure did not show an obviously positive effect on cell proliferation, differentiation, and mineralization. By contrast, combining a hydrophilic nano/submicro-scale pore structure with biomolecule immobilization greatly enhanced cell growth with a particularly pronounced effect on initial cell differentiation and cell mineralization.

Using the natural cross-linker genipin to immobilize type I collagen on sandblasted and alkaline-etched Ti surfaces resulted in a surface feature with a hydrophilic nano/submicro-scale pore network. The proposed surface modification scheme enhanced the initial cell–surface interaction, resulting in accelerated cell proliferation, initial cell differentiation, and mineralization.

## 5. Conclusions

In the current study, the natural cross-linker genipin was used to immobilize type I collagen on Ti surfaces pre-treated using sandblasting and alkaline etching. There were no significant differences in micrometer-scale surface roughness among the test Ti surfaces. The nano/submicro-scale pore network and hydrophilic characteristics of the sandblasted rough Ti surfaces did not have a positive effect on cell adhesion, with corresponding negative effects on cell proliferation, initial differentiation, and mineralization. In the absence of type I collagen, hydrophilic rough Ti surfaces with or without a nano/submicro-scale pore network were insufficient to promote cell differentiation or mineralization. Combining the nano/submicro-scale pore network and hydrophilic surface with type I collagen immobilization on rough Ti surfaces greatly enhanced hMSCs adhesion, proliferation, and particularly initial differentiation and mineralization.

## Figures and Tables

**Table 1:** Surface roughness of test samples presented as Sa and Sq (μm) (groups with different letters were significantly different (*p* < 0.05).

	Sa	Sq
**SBTi**	1.10 ^A^ ± 0.05	1.27 ^A^ ± 0.06
**SBTiOH**	0.91 ^B^ ± 0.02	1.13 ^A^ ± 0.02
**SBTiG**	0.93 ^B^ ± 0.04	1.21 ^A^ ± 0.05
**SBTiGC**	0.87 ^B^ ± 0.09	1.14 ^A^ ± 0.08

**Table 2:** ELISA analysis results, indicating the OPN expression level and mean ± SD (pg/mL) of test Scheme 7. and 14 d (groups with different letters are significantly different (*p* < 0.05).

	SBTi	SBTiOH	SBTiG	SBTiGC
**7 d**	70.89 ^a^ ± 13.10	25.43 ^b^ ± 14.81	14.97 ^b^ ± 5.23	99.37 ^a^ ± 12.85
**14 d**	123.80 ^a^ ± 22.73	39.82 ^b^ ± 13.31	32.23 ^b^ ± 9.96	241.31 ^c^ ± 41.15

## Data Availability

The data that support the findings of this study are available from the corresponding author upon reasonable request.
